# Supplementary material for: Crystal structure of lipid A disaccharide synthase LpxB from Escherichia coli
Source: Nat Commun. 2018 Jan 25;9:377. doi: 10.1038/s41467-017-02712-9 (PMC5785501; doi:10.1038/s41467-017-02712-9)
Supplement: Supplementary file 1 — Supplementary Information [file 41467_2017_2712_MOESM1_ESM.pdf]

Supplementary Information

<u>Crystal Structure of Lipid A Disaccharide Synthase LpxB from *Escherichia coli*</u>

Bohl et al.

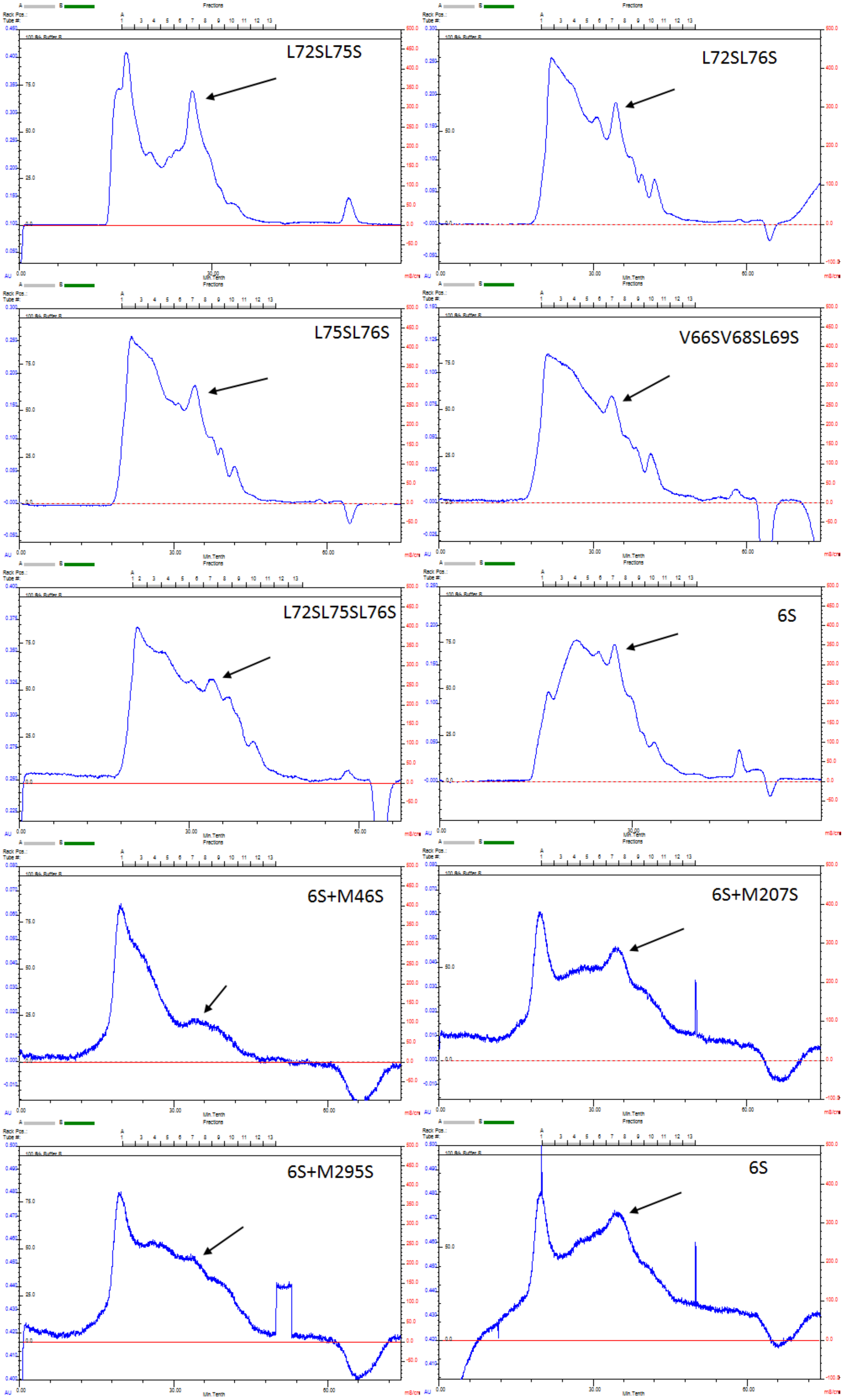
L72SL75S
L72SL76S
L75SL76S
V66SV68SL69S
L72SL75SL76S
6S
6S+M46S
6S+M207S
6S+M295S
6S

Supplementary Figure 1: Size exclusion chromatography. Ni-NTA purified LpxB mutants were run on 10/300 GL Superdex 200 columns (GE Healthcare). The top 6 UV traces are from protein run on an "Increase" model column at 0.5 mL/min. The bottom 4 UV traces are from protein run on the standard model column. Crystallizable LpxB appeared as a peak in fractions 7-8 (indicated by arrows). Large amounts of LpxB ended up in the void/soluble aggregate fractions preceding fraction 7. The quality of various mutants was judged by the peak heights of the aggregated and fraction 7-8 peaks. LpxB6S has relatively little protein in the void peak compared to soluble forms with fewer mutations. M207S preserves the behavior of LpxB6S while M46S and M295S lead to more protein aggregation.

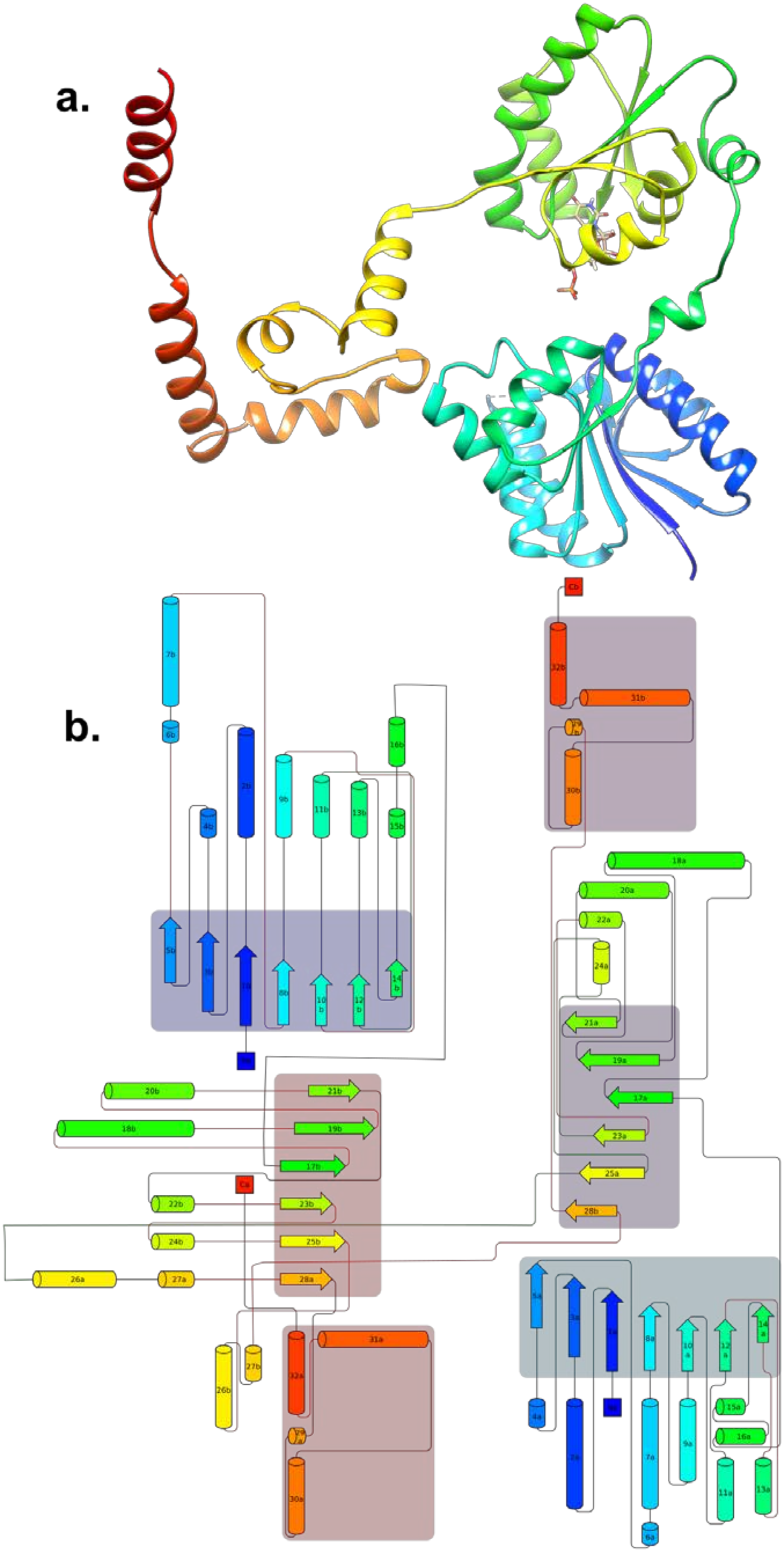
a.
b.

Supplementary Figure 2: LpxB forms an intertwined dimer. A. shows the isolated UDP-bound LpxB monomer. **B.** shows the 2D depiction of the LpxB dimer generated with Pro-origami[1]. The rainbow coloring of secondary structural elements is similar to that in **A**, and the numbering of secondary structural elements is sequential for each subunit (a and b). Colored boxes indicate strands (arrows) in the same sheet and the final helices (cylinders) that wrap around the bottom of the other subunit.

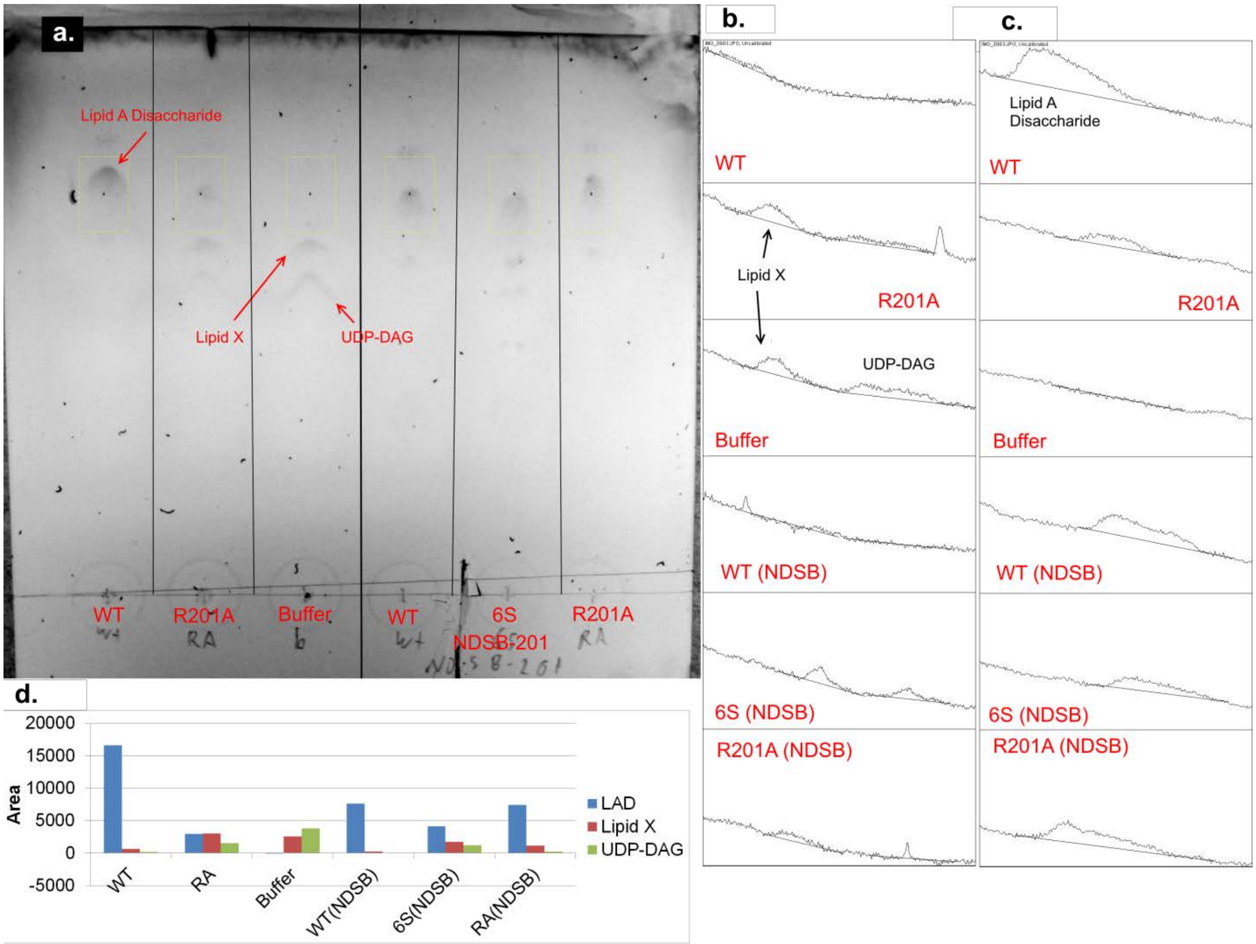

Supplementary Figure 3: Activity of LpxB6S mutant. A. Products from overnight (18.75 hr) reactions under standard conditions (left 3) or with 0.9 M NDSB 201 (right 3) were run on a Silica gel 60 HPTLC plate. Charring shows product in the all lanes except the no enzyme control. Lipid X is visible in all lanes, and UDP-DAG is visible in the left LpxBR201A lane, the LpxB6S lane, and the negative control lane. These results were replicated once. The intensity of the bands in **A** were analyzed in ImageJ[2]: defined lanes were plotted as distance versus intensity, and the area under the peak was determined. **B.** shows the curves for the reactant bands, and **C.** shows the curves for the product band. **D.** shows a graphical depiction of the areas under the peaks in **B** and **C**.

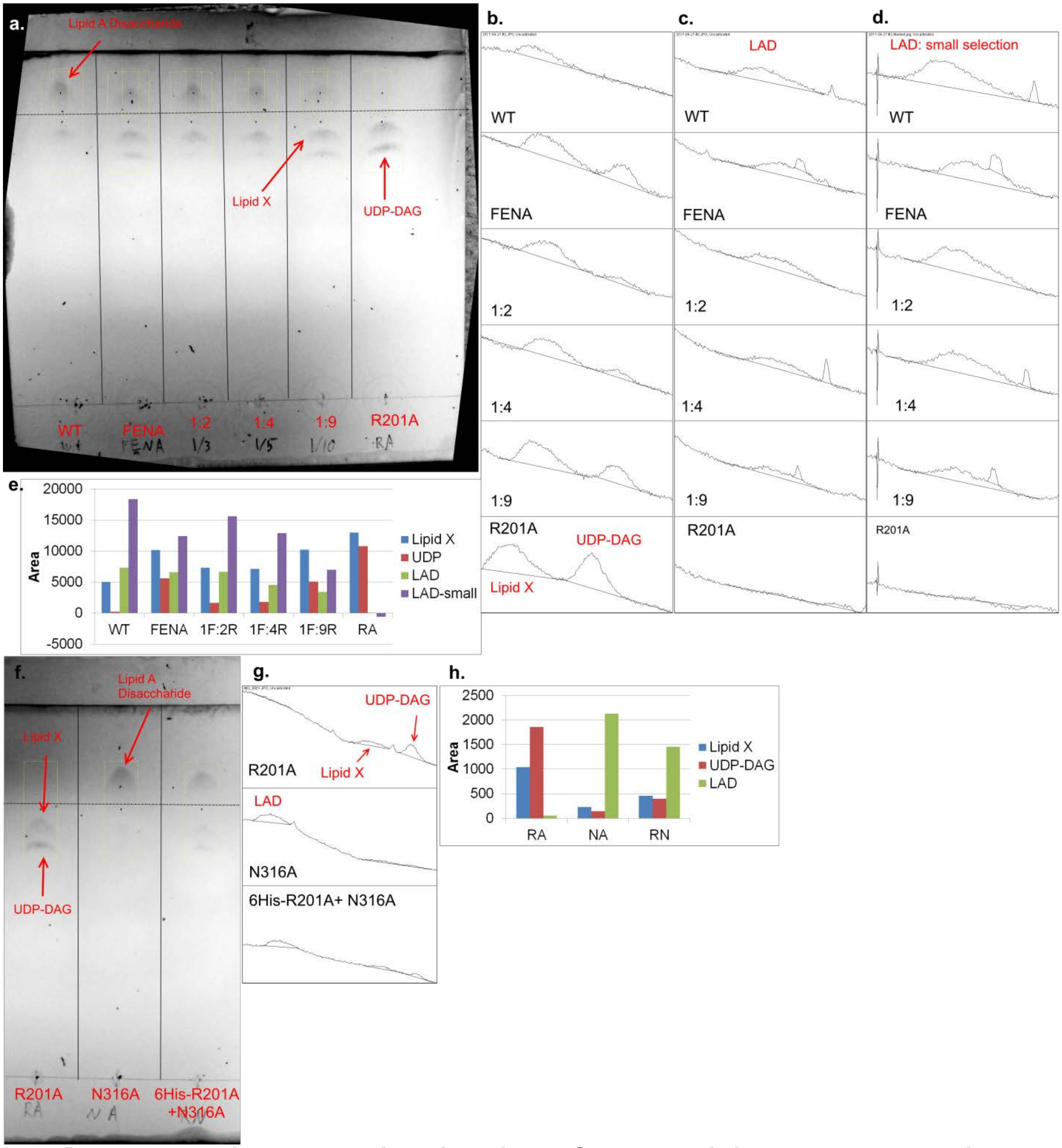

Supplementary Figure 4: Dimerization of R201A with F298AN316A and N316A. **A.** Three hour reactions included wild-type LpxB, LpxBR201A, and LpxBFN combined in shown ratios with LpxBR201A (constant total protein). Consistent with the UDP-release assays, UDP-DAG bands are fainter and product bands appear darker in 1:2 and 1:4 lanes than in the 100% LpxBFN lane. The increase in activity of the 1:2 mixture relative to 100% LpxBFN was replicated 4 times. TLC analysis of the 1:4 and 1:9 mixtures was not repeated. **B.** Reactant band intensity was analyzed in ImageJ[2]: lanes were plotted as distance versus intensity and the areas under the peaks were determined. **C.** The same analysis of product bands. The irregular shape of these bands gives poor peak resolution, making quantification more

difficult. **D.** When only the central, darkest potion of the product bands is selected, peak resolution is improved. **E.** The quantification of the peak areas. The decrease in lipid X and UDP-DAG intensities 1:2 and 1:4 mixtures support the increase in activity produced by combining LpxBFN and LpxBR201A observed in UDP-release assays. As in the UDP-release assays, activity appears to decrease with increasing percent LpxBR201A. When the product band intensity is quantified with the smaller, central selection, the wild-type and 1:2 mixture lanes show a corresponding increase in product band intensity. However, when the full band was selected, there was little difference between wild-type, LpxBFN, and the 1:2 mixture, and the 1:4 mixture even showed decreased product band intensity. The 1:9 mixture shows weaker product band intensity in either case. Regardless, these data support the conclusion from the UDP-release assays that LpxBFN and LpxBR201A form a complementary dimer. **F.** One hour reactions of LpxBR201A, LpxBN316A, and co-purified His-tagged LpxBR201A with untagged LpxBN316A. While LpxBR201A did not produce a detectable amount of product, LpxBN316A and the co-purified proteins show production of lipid A disaccharide with corresponding depletion of reactants indicating that LpxBR201A can pull down LpxBN316A. These results were replicated five times. **G. H.** Analysis was performed as for the plate in **A.**

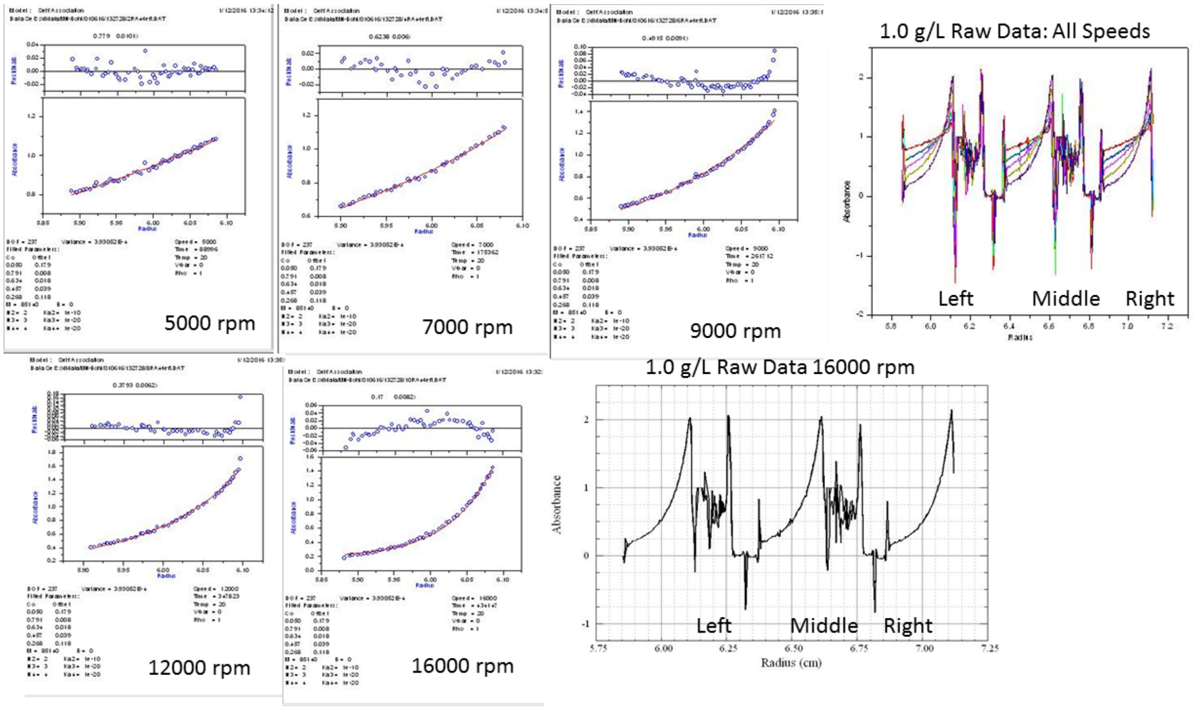

Supplementary Figure 5: Analytical ultracentrifugation. Raw and fitted analytical ultracentrifugation data are shown. LpxB6S (120 μL) was centrifuged at 5000, 7000, 9000, 12000, or 16000 rpm for 10 hours in a ProteomeLab XL-A ultracentrifuge. Absorbance was measured at 280 nm. Fitting of distance versus absorbance data is shown for the left wells. Fitting with a solvent density of 1.0 g/mL and a protein partial volume of 0.73 mL/g gave a protein mass of 71-79 kDa, most consistent with the expected 84.5 kDa dimer.

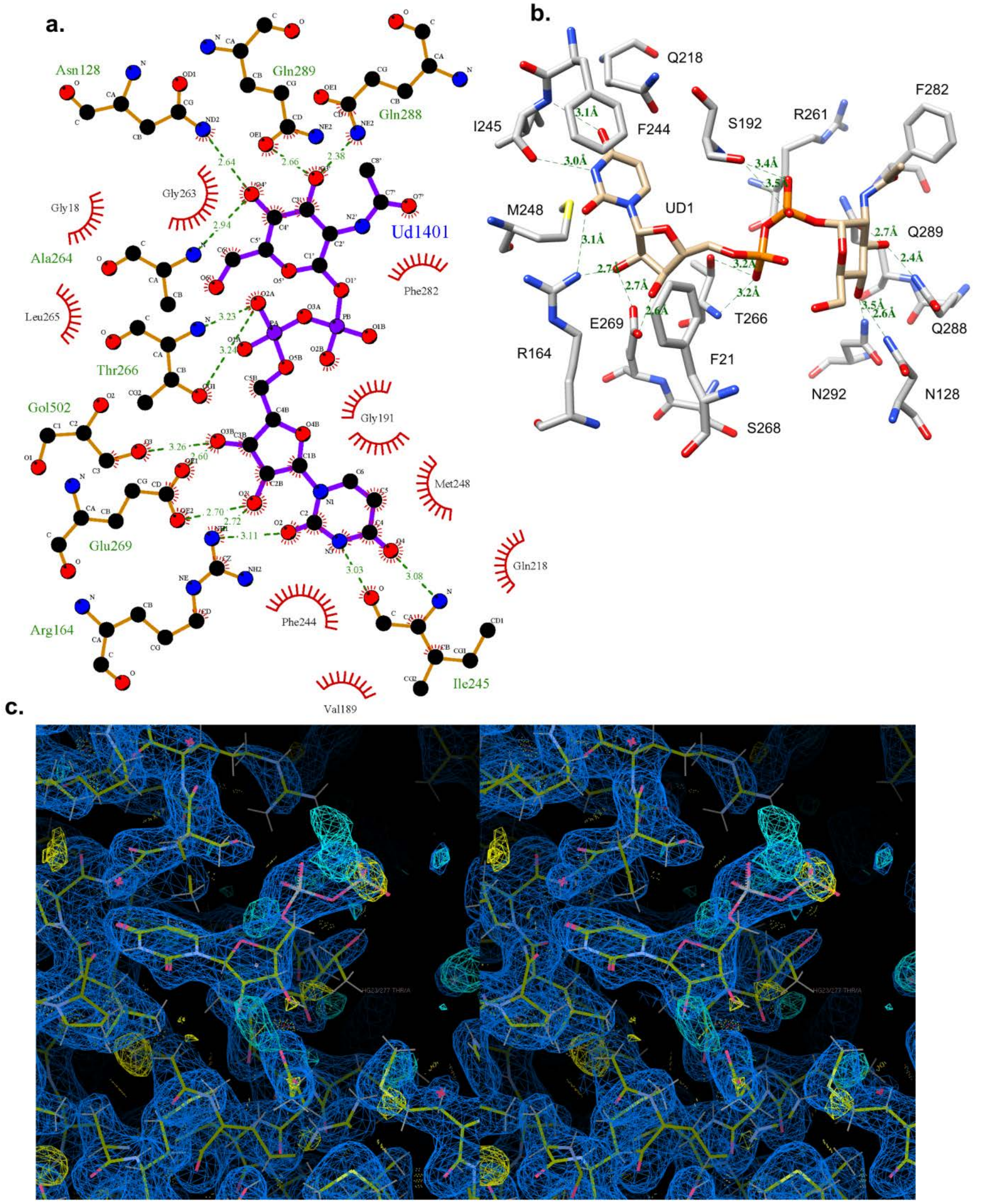

Supplementary Figure 6: MurG active site. A. B. The MurG active site from PDB entry 1NLM[3] is provided for comparison to Figure 3. **C.** Stereo view of LpxB UDP-binding pocket. The 2mFo-DFc map (blue mesh) is shown at 0.4317 e/Å^3 (0.99 rmsd), and the mFo-DFc map (cyan mesh for positive and yellow mesh for negative) is shown at 0.45 e/Å^3 (3.01 rmsd).

```
MurG/1-364       1 MMSGQG..........................KRLMVMA.G.....GTGG
PimA/-19-386   -19 ............MGSSHHHHHHSSGLVPRGSHMRIGMVCPYSFDVPG..G
LpxB/1-382       1 ......MTEQRP...................LTIALVAGE.....T..S
                                                    : ::.         .

MurG/1-364      19 HVFPGLAVAHHLM.A..Q.GWQVRWLGTADRMEA.DLVPKHGI.EIDFI.
PimA/-19-386    17 VQSHVLQLAEVLRDA....GHEVSVLAPA...SPHVK.L..PD.YVVSG.
LpxB/1-382      18 GDILGAGLIRAL..KEHVPNARFVGVA.....G..PRMQ.AEGCEAWYEM
                          :  .  *       .  ..   :.

MurG/1-364      62 R.IS............................GLRGKGIKALIAAPLRIFNAWR
PimA/-19-386    55 GK.A...........VPIPYNGSVARLRF............G..PATHR
LpxB/1-382      58 EE.LAVMGISESSGR.............S.............RRSSHIRA

MurG/1-364      87 QARAIMKAYKPDVVLGMGG...YVSGPGGLAAWSLGIPVVLHEQNGI...
PimA/-19-386    78 KVKKWIAEGDFDVLHIHEPNAPSLSMLALQAAE...GPIVATFHT.STTK
LpxB/1-382      81 DLTKRFGELKPDVFVGIDAP.DF.NITLEGNLKKQGIKTIHYVS..P...
                   .    :   . **.          .             :

MurG/1-364     131 .AGLTN..K....W.LAK..IATKVMQAFPGA..FPN......AEVVGNP
PimA/-19-386   124 SLT...LSVFQGILRPYHE.KIIGRIAVSDLARRWQMEALGSDAVEIPNG
LpxB/1-382     124 .SVW....AWRQKRVFKIGRATDLVLAFLPFEKAFYDK.YNVPCRFIGHT
                                             :        :        .  :  :

MurG/1-364     163 ..VRTD...VLALPLPQQRLAGREGPVRVL..............VVGGSQ
PimA/-19-386   170 ..VDVASF.A...................DAPLLDGYPREGRTVLFLGR
LpxB/1-382     168 MADA....MP.....L...D.......PDK...................

MurG/1-364     194 G.....ARILNQTMPQVAAKLGDSVTIWH..QSGKGSQQSVEQAYAEAGQ
PimA/-19-386   197 YDEPRKGMAVLLAALPK.......................L...V...AR
LpxB/1-382     179 ..........................NAARDVL........G..I....P

MurG/1-364     237 PQH.KVTE..........................................
PimA/-19-386   218 FP.DVEILIVG.......................................
LpxB/1-382     189 ..H........DAHCLALLPGSRGAEVESLSADFLKTAQLLRQTYPDLEI

MurG/1-364     244 .................................................F
PimA/-19-386   228 ....RGD.ED.E...LRE...........QAGDLAGHLRF.LGQVDDATK
LpxB/1-382     229 VVPLVNAK..RREQFE..RIKAEVAPDLSVH...L..L.DG........M

MurG/1-364     245 ..IDD...............................MA..AAYAWADVV
PimA/-19-386   257 ASAMRSADVYCA.P.....H.L...GG.ESFGIVLVEAMAAGTA..V..V
LpxB/1-382     261 .........GREAMVASDAALLASGTAAL..E.................
```

```
MurG/1-364     259 VCRSG.ALTV.SEIAAAG....L.PALFVPFQ......... 283
PimA/-19-386   292 ASDL.DA..FRR..V..LADGDAGRLV.....P.V...... 313
LpxB/1-382     282 .CM......L.......S.......KC.....P.MVVGYRM 295

MurG/1-364     284 HKDR.QQY.WNA..........LPLEK.A...GAAKII.EQP...QLSV.
PimA/-19-386       ..................................................
LpxB/1-382     296 KPFTF...WL..AKRLVKTDYVSLPNLLAGRELV.KELL...QEEC...E

MurG/1-364     313 D..AVAN.TLA.G..W..SRETLLTMAE.R..ARAAS..IPDATERVANE
PimA/-19-386   346 ....................................RYDWSVVSAQIMR
LpxB/1-382     334 PQKL..AA...ALLPLLANGKTSHAMHDTFRELHQQI..RCNADEQAAQA
                                                                .. .

MurG/1-364     350 VSRVARALEHHHHHH..................... 364
PimA/-19-386   359 VYETVS.G.......AGIKVQVSGAANRDETAGESV 386
LpxB/1-382     377 VLELAQ.............................. 382
                   *  . .
```

Supplementary Figure 7: Structural alignment of LpxB (PDB: 5W8S), MurG (PDB: 1F0K), and PimA (PDB: 2GEJ). Purple arrows and blue ovals show the positions of β-sheets and α-helices in the LpxB sequence. Yellow highlighting marks the disordered loops in LpxB and PimA, and green highlighting marks the amphipathic helices involved in membrane association[4,5]. Magenta highlighting marks helices 14 and 15 in LpxB which contain F298 and N316, respectively. Cyan highlighting marks the final 3 helices in LpxB. Red lettering marks the locations of point mutations made in LpxB.

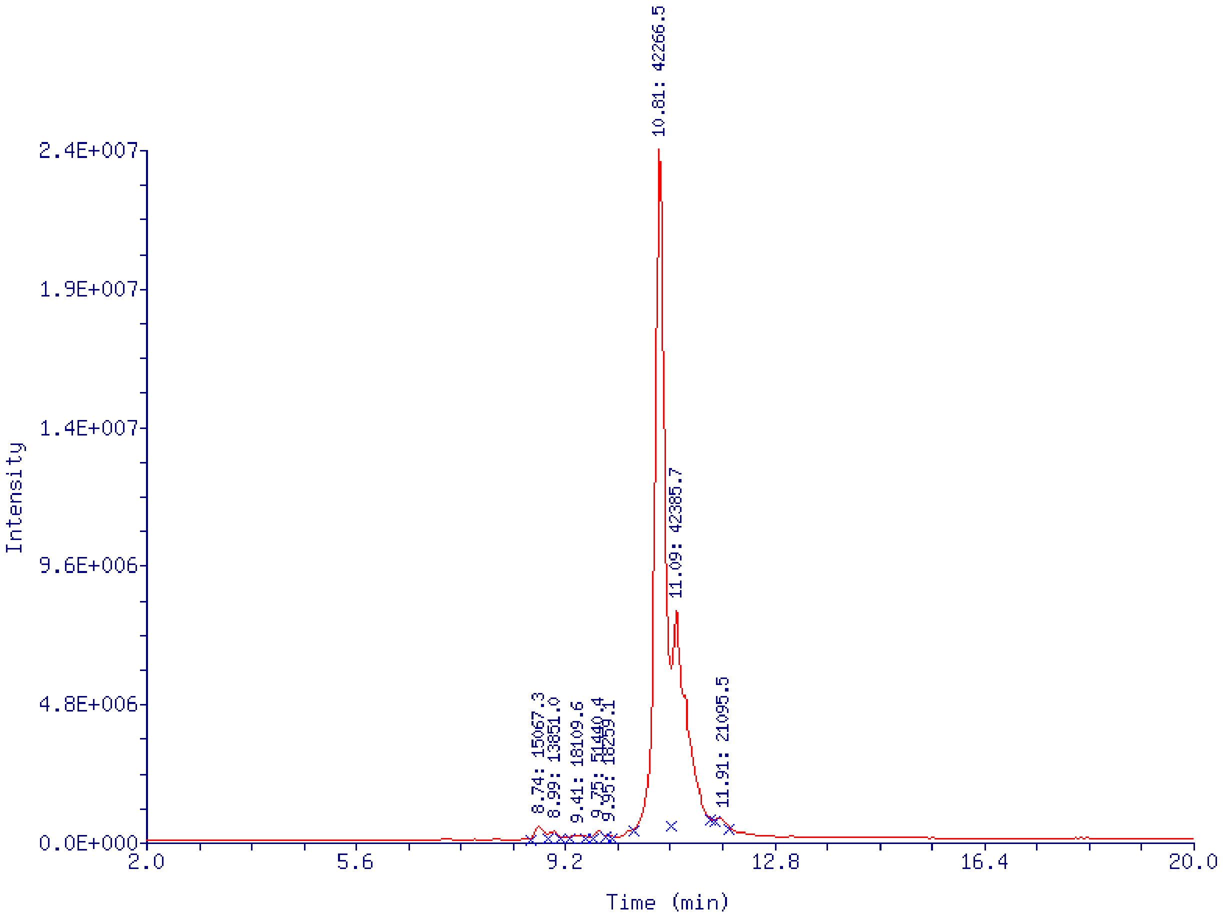

Supplementary Figure 8: Liquid chromatography-mass spectrometry. LCMS of LpxB6S gives a peak at an m/z of 42266.5, which corresponds well with the calculated molecular mass of 42270.01 Da from the ProtParam server <http://web.expasy.org/protparam/>. A second peak at 42385.7 (119.2 Da larger) probably corresponds to a cyteinylated form of LpxB. Indeed, electron density at C367 of the unbound LpxB7S structure suggests some modification (data not shown). This is likely a result of oxidation stress in the *E. coli.*

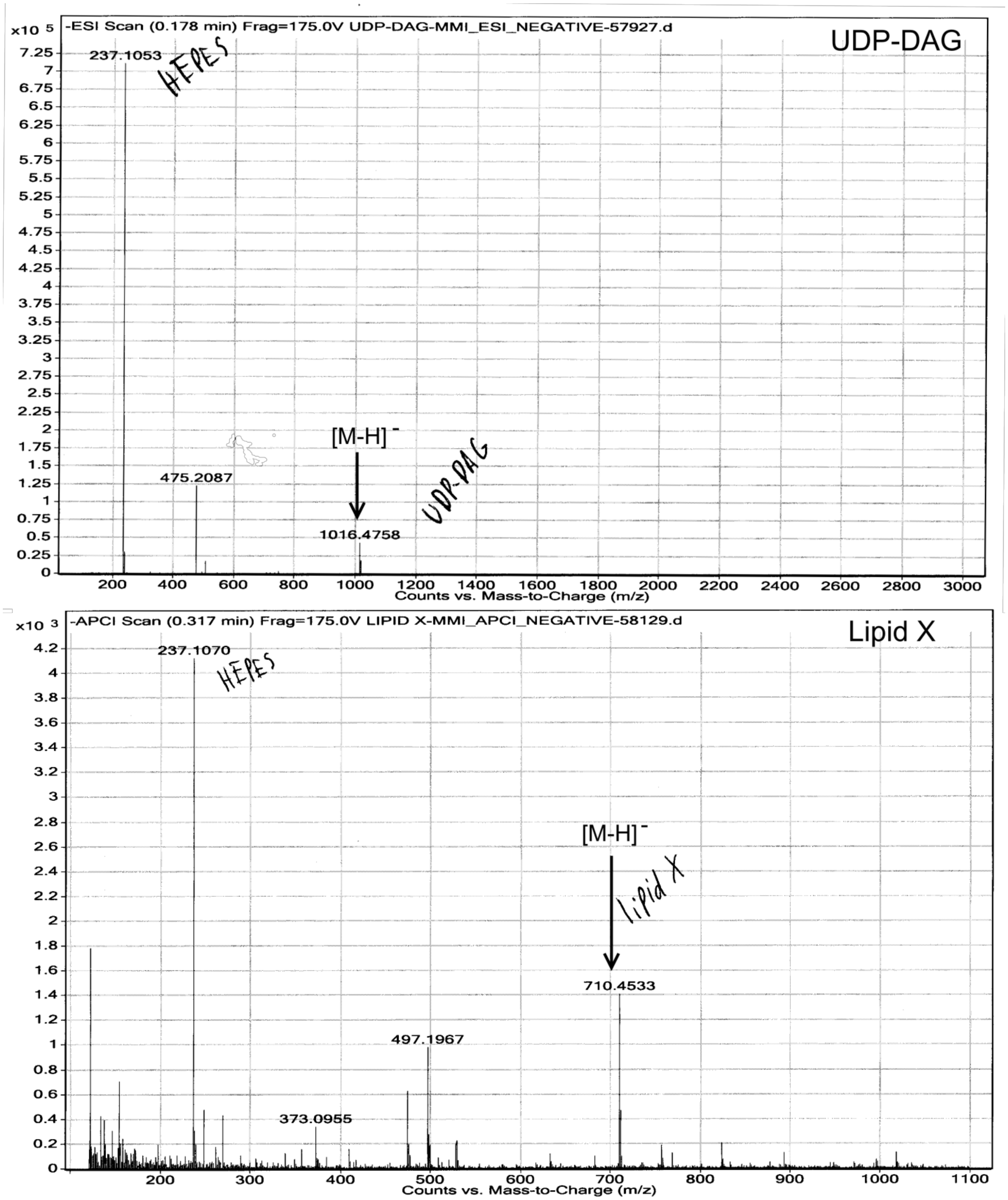

Supplementary Figure 9: Mass spectra of LpxB reactants. UDP-DAG and Lipid X were extracted from CcLpxI, wild-type and D225A respectively[6]. The identity of the purified lipids was confirmed by their $[M-H]^-$ peaks, which correspond well with the predicted exact masses of lipid X (711.43 Da) and UDP-DAG (1017.46 Da). The mass spectra were collected in negative ion

mode with direct infusion of the diluted lipids and ionization by electrospray or atmospheric pressure chemical ionization.

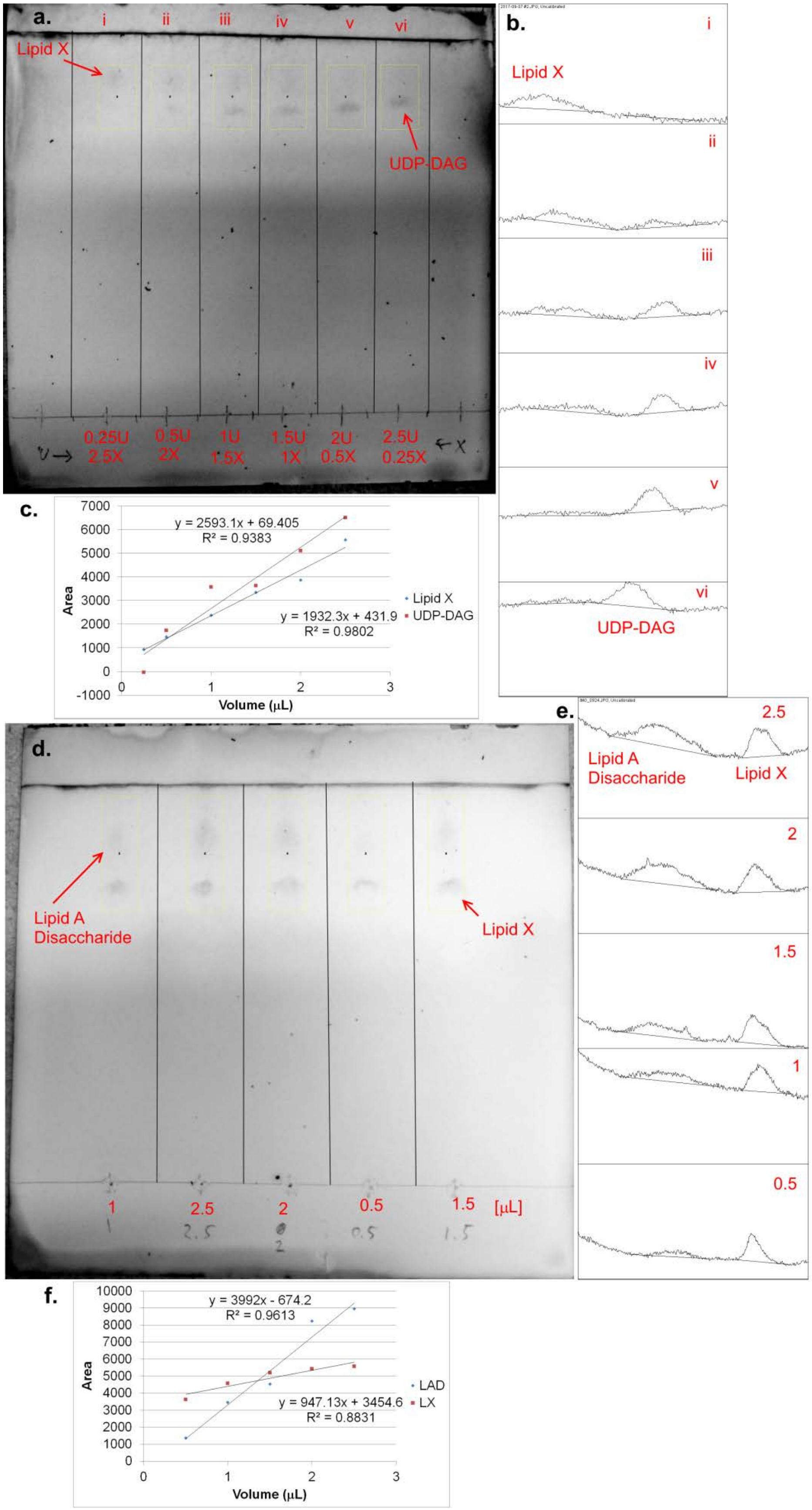
a.
i
ii
iii
iv
v
vi
Lipid X
UDP-DAG
0.25U 2.5X
0.5U 2X
1U 1.5X
1.5U 1X
2U 0.5X
2.5U 0.25X
b.
i
Lipid X
ii
iii
iv
v
vi
UDP-DAG
c.
y = 2593.1x + 69.405
R² = 0.9383
y = 1932.3x + 431.9
R² = 0.9802
Lipid X
UDP-DAG
Area
Volume (μL)
7000
6000
5000
4000
3000
2000
1000
0
-1000
0
1
2
3
d.
Lipid A Disaccharide
Lipid X
1
2.5
2
0.5
1.5
[μL]
e.
2.5
Lipid A Disaccharide
Lipid X
2
1.5
1
0.5
f.
y = 3992x - 674.2
R² = 0.9613
y = 947.13x + 3454.6
R² = 0.8831
LAD
LX
Area
Volume (μL)
10000
9000
8000
7000
6000
5000
4000
3000
2000
1000
0
0
1
2
3

Supplementary Figure 10: Linear relationship between lipid amount and band intensity. A. Different volumes [µL] of UDP-DAG (0.31 mM) and lipid X (~0.31 mM) were run on an HPTLC Silica gel 60 plate. Once dry, the plate was charred with 20% sulfuric acid in ethanol and a heat gun. This plate was replicated once. **B.** Band intensities were analyzed in ImageJ[2]: lanes were plotted as distance versus intensity and the areas under the peaks were determined. **C.** Volumes run were plotted versus peak areas, and the data were fitted by a line in MS Excel. Both reactants show increasing band intensity with increasing lipid amount, and the relationships are quite linear: R^2 of 0.9802 for lipid X and 0.9383 for UDP-DAG. **D.** Different volumes [µL] of UDP-DAG (0.31 mM) and lipid X (1.3 mM) were reacted to completion with 0.5 g/L wild-type LpxB in 0.1 M Tris-HCl pH 8.0, 0.1% Triton X-100, and 1 mg/mL BSA at 30°C for 1 hr. TLC of products was performed as in **A.** This plate was not repeated. **E.** Band intensities of lipid A disaccharide prouct and remaining lipid X were analyzed as in **C. F.** Volumes reacted were plotted versus the band intensities, and the data were fitted by a line in MS Excel. Band intensities increase with increasing lipid amounts. The lipid A disaccharide data are quite linear (R^2 of 0.9613), and the lipid X data are reasonably linear (R^2 of 0.8831).

Supplementary Table 1: LpxB melting temperature.

LpxB	T_m (^{o}C)
V66SV68SL69S (VVL)	49.0 ± 0.4
VVL-R201A	49.5 ± 0.4
VVL-N316A	49.2 ± 0.5
VVL-F298EN316A	48.5 ± 0.0

Melting was observed by SYPRO Orange fluorescence. T_m values are the temperatures at the inflection points of the melting curves plus or minus the standard deviations (n= 4).

Supplementary Table 2: Semi-quantitative comparison of LpxB activities by TLC.

LpxB	Time to Reaction Completion
wild-type	1 min<t<5 min
L72SL75S	10 min<t<30 min
L72SL76S	1 min<t<5 min
L75SL76S	1 min<t<5 min
V66SV68SL69S (VVL)	1 hr<t<2 hr
L72SL75SL76S (LLL)	5 hr<t< 6 hr
6S	18.75 hr*<t
N316A	30 min<t<1 hr
F298EN316A	15.5 hr[‡]<t
R201A	18.75 hr<t
6SR201A	No activity detected

Reactions were performed with 0.5 mg/mL LpxB, 0.11 mM UDP-DAG, ~0.13 mM lipid X, 0.5 mg/mL BSA, 0.05-0.06% Triton X-100 and 0.1 M Tris-HCl pH 8 at 30°C. *LpxB6S showed no activity under standard conditions but was active when 0.9 M NDSB 201 was added. ‡Reactions with LpxBFN appeared mostly complete by 4.5 hr, but were never observed to go to completion.

Supplementary References

1. Stivala, A., Wybrow, M., Wirth, A., Whisstock, J. C. & Stuckey, P. J. Automatic generation of protein structure cartoons with Pro-origami. *Bioinformatics* **27,** 3315–3316 (2011).
2. Schneider, C. A., Rasband, W. S. & Eliceiri, K. W. NIH Image to ImageJ: 25 years of image analysis. *Nat. Methods* **9,** 671–675 (2012).
3. Hu, Y. *et al.* Crystal structure of the MurG:UDP-GlcNAc complex reveals common structural principles of a superfamily of glycosyltransferases. *Proc. Natl. Acad. Sci. U. S. A.* **100,** 845–849 (2003).
4. Guerin, M. E. *et al.* Molecular recognition and interfacial catalysis by the essential phosphatidylinositol mannosyltransferase PimA from mycobacteria. *J. Biol. Chem.* **282,** 20705–20714 (2007).
5. Rodrigo-Unzueta, A. *et al.* Molecular Basis of Membrane Association by the Phosphatidylinositol Mannosyltransferase PimA Enzyme from Mycobacteria. *J. Biol. Chem.* **291,** 13955–13963 (2016).
6. Metzger, L. E., 4th, Lee, J. K., Finer-Moore, J. S., Raetz, C. R. H. & Stroud, R. M. LpxI structures reveal how a lipid A precursor is synthesized. *Nat. Struct. Mol. Biol.* **19,** 1132–1138 (2012).
